# Cohort survey of college students’ eating attitudes: interventions for depressive symptoms and stress coping were key factors for preventing bulimia in a subthreshold group

**DOI:** 10.1186/s13030-018-0127-y

**Published:** 2018-05-24

**Authors:** Yuri Okamoto, Yoshie Miyake, Ichie Nagasawa, Masaharu Yoshihara

**Affiliations:** Health Service Center, Hiroshima University, 1-7-1, Kagamiyama, Higashihiroshima, Hiroshima, 739-8514 Japan

**Keywords:** Eating disorder, Cohort, College students, Subthreshold, Depressive symptoms, Stress coping, Prevention

## Abstract

**Background:**

The aim of this study was to examine the necessity of early intervention for students with potential bulimia by investigating how the eating attitudes of college students change and examining the relation between bulimic symptoms and depressive symptoms or the ability to cope with stress.

**Methods:**

The study participants were students who entered Hiroshima University in 2014. This study was conducted at two time points: Time-1 in 2014 and Time-2 in 2017. The Eating Attitudes Test-26 (EAT-26), Bulimic Inventory Test, Edinburgh (BITE), Beck Depression Inventory-II (BDI-II), and Coping Inventory for Stressful Situations (CISS) were administered at Times 1 and 2, and the responses were compared between the time points. Next, we compared the BDI-II scores of the BITE improved and worsened groups. In addition, we divided the participants into a clinical group, subthreshold group, and healthy group based on the BITE score at Time-1to compared their depressive symptoms and the ability to cope with stress.

**Results:**

Significantly higher BITE and BDI-II scores were recorded for both males and females at Time-2 than at Time-1. The BDI-II score at Time-1 was significantly higher in the BITE worsened group than in the BITE improved group. The BDI-II scores at Time-1 were significantly higher for both males and females in the subthreshold group than in the healthy group. Furthermore, significantly higher CISS-T and CISS-E scores were recorded at Time-1 for females in the subthreshold group than for females in the healthy group.

**Conclusions:**

Based on these results, intervention for students the subthreshold group is important, and the key to intervention may be to address not only eating behaviors but also depressive symptoms and stress coping.

**Trial registration:**

UMIN000029474 Registered 9 October, 2017 (retrospectively).

## Background

Eating disorders are serious illnesses that can be difficult to treat [1]. Many serious problems are associated with eating disorders [2, 3], such as 1) significant mortality rates [4], 2) suicidal behavior [5, 6], 3) high medical costs [7], 4) high rates of comorbidity [8], and 5) strong associations with childhood trauma, such as abuse. Furthermore, long-term treatment for eating disorders is often required, and many prolonged cases have been observed. Eating disorders often develop in adolescence, resulting in a loss of important time for adolescents to make career decisions and prepare for an active role in society. Therefore, strategies that prevent eating disorders and early interventions are important. In particular, bulimia nervosa (BN) is highly comorbid with other conditions, such as depression, impulsive behavioral problems, and suicide [4]. Early intervention, particularly for BN, is therefore an important task to be addressed. However, clear answers as to when and how to provide early interventions are still unavailable.

The aim of this study was to examine the necessity of early intervention by investigating how the eating attitudes of college students change and the relation between bulimic symptoms and depressive symptoms or stress coping. We chose college students as the subjects, because it has been reported that the onset of both anorexia nervosa (AN) and BN increases from adolescence through the college period [9, 10]. We focused on students who showed abnormal eating behaviors, particularly after entrance to college. In Japan, students in college may experience a mental health crisis [11, 12]. In late adolescence through young adulthood, youth are expected to rapidly form their identities and are exposed to psychosocial risk factors such as academic stress and relationship troubles, which are associated with psychiatric difficulties [13].

Regarding eating disorders, the first year of college has been suggested as a critical time period for the development of disordered eating [14, 15]. Many reports suggest that effective prevention, identification, and treatment services are needed to address the observed increase in the incidence of eating disorders on campus [16–19]. As a result, the risk of eating disorders is thought to be increased in college students.

Although AN may be suspected due to low body weight, BN does not result in low body weight and thus cannot be easily determined based on an outward physical manifestation. Therefore, it is important to study intervention methods aimed at targeting the early stage of bulimia. In the present study, we studied eating attitudes at two time points, university entrance and the fourth year, and observed how college students’ eating attitudes changed over time. Some research reported that subthreshold cases often progress to threshold cases, and early intervention has been shown to prevent severe illness [20–22]. Interventions for depression are important because individuals with eating disorders are often suicidal. In addition, we previously reported on the relationships between eating disorders, depressive symptoms and stress coping, and the results showed improvements following treatment [23, 24]. We hypothesized that partial symptoms of bulimia would appear early and would be related to depressive symptoms; in addition, this tendency would be noticeable in female students.

Because this study is not anonymous, we were able to observe the changes of individual students, which represents a new point that is not found in previous reports.

## Methods

### Procedure

We surveyed college students’ eating attitudes, depressive symptoms, and stress coping using self-assessment scales conducted at the time of the admission medical examination (Time-1) and at the time of the 4th year medical examination (Time-2). We compared eating attitudes, depressive symptoms, and stress coping between the two time points.

Next, we divided the participants into a clinical group, a subthreshold group, and a healthy group using the Bulimic Inventory Test, Edinburgh (BITE) score at Time-1. The cut-off point for the BITE score is 20 points; therefore, the clinical group had a score of 20 or more, the subthreshold group had a score ranging from 10 to 19, and the healthy group had a score of 9 or less. Then, we followed each student to determine the change from Time-1 to Time-2. In addition, we compared the depressive symptoms and stress coping of the group whose BITE score improved from Time-1 to Time-2 and the group whose BITE score worsened from Time-1 to Time-2.

Furthermore, to identify risk factors, we compared the depressive symptoms and stress coping of the subthreshold and healthy groups.

### Participants

The participants in this study were students who entered Hiroshima University in 2014. This study was conducted at two points: Time-1, 2014 (the time at which the students entered college), and Time-2, 2017 (in the 4th year). The participants included 620 students (330 male, and 290 female, aged 21.3 ± 0.9 years) whose characteristics are shown in Table 1.

**Table 1:** Characteristics of participants

	Time-1	Time-2
Males	N = 977	N = 330
Age (years)	20.0 ± 2.1	22.6 ± 3.4
BMI (kg/m^2^)	21.0 ± 2.8	21.3 ± 3.1
Females	*N* = 867	*N* = 290
Age (years)	19.0 ± 2.5	22.3 ± 0.9
BMI (kg/m^2^)	20.0 ± 2.5	20.6 ± 2.4

### Measures

#### Eating attitudes test-26 (EAT-26)

The EAT-26 is a 26-item self-report measure of eating attitudes [25]. The original scale consists of 40 items (EAT-40), which was shortened to the EAT-26 by Garner, who reported that the EAT-26 score is highly correlated with the EAT-40 score. It is a reliable, valid and economical instrument. Answers are provided on a six-point scale ranging from “not at all” to “extremely”. The cut-off point is 20 points, and scores greater than 20 indicate a high possibility of an eating disorder.

#### Bulimic inventory test, Edinburgh (BITE)

The BITE is a 36-item self-report measure of bulimic symptoms [26] scored on a six-point scale ranging from “not at all” to “extremely”. It consists of a symptom evaluation scale (30 items) and a severity scale (6 items). The cut-off point is 20 points.

#### Beck depression inventory-II (BDI-II)

The BDI-II is a 21-item self-report measure [27] scored on a 4-point scale. The cut-off points are: 0–9 indicates that a person is not depressed, 10–18 indicates mild-moderate depression, 19–29 indicates moderate-severe depression, and 30–63 indicates severe depression.

#### Coping inventory for stressful situations (CISS)

The CISS was developed by Endler [28]. It is a 48-item self-report measure scored on a five-point scale ranging from “not at all” to “extremely”. This scale evaluates three coping behavior patterns: task-oriented, emotion-oriented, and avoidance-oriented coping. The CISS is composed of the following three scales: task-oriented coping: solving the problem, cognitive restructuring of the problem, or attempts to alter the situation; emotion-oriented coping: reactions include emotional responses; and avoidance-oriented coping: activities and cognitive changes to avoid the stressful situation. Two additional subscales for avoidance-oriented coping have been reported, but only the three main scales were considered in the present study.

### Data analysis

SPSS (IBM Corporation, Armonk, NY) version 21 was used for the statistical analyses. We used the paired t-test to compare the EAT, BITE, BDI, and CISS scores of Time-1 and Time-2. The Kruskal-Wallis test was used to compare the scores of the improved, worsened, and unchanged groups. In addition, we used the Mann-Whitney test to compare the scores between the subthreshold group and the healthy group. *P* < 0.05 indicated a significant difference.

## Results

### Changes in the EAT, BITE, and BDI scores from Time-1 to Time-2

We investigated changes in the EAT-26, BITE, BDI-II, and CISS scores recorded by male and female students from Time-1 to Time-2 (Tables 2 and 3). Significantly lower EAT-26 scores were recorded by males at Time-2 than at Time-1, and the scores for females tended to decrease at Time-2. Significantly higher BITE scores were recorded for both males and females at Time-2 than at Time-1. Both males and females recorded significantly higher BDI-II scores at Time-2 than at Time-1. None of the subscale scores of the CISS were significantly different at Time-1 and Time-2 between males and females. Furthermore, we examined the correlation between BITE and BDI-II scores; positive correlations were observed for males at both Time-1 (*r* = 0.440, *P* < 0.000) and Time-2 (*r* = 0.438, P < 0.000) and for females at both Time-1 (*r* = 0.462, P < 0.000) and Time-2 (*r* = 0.511, P < 0.000). A significant correlation between BITE and CISS scores was not observed.

**Table 2:** Male EAT, BITE, BDI, CISS scores at Times1 and 2

	Time-1	Time-2	*P*-value
	N = 330	N = 330	
EAT-26	2.7 ± 3.5	2.1 ± 3.1	0.006**
BITE	4.2 ± 3.4	4.7 ± 4.0	0.002**
BDI-II	5.3 ± 5.1	7.6 ± 6.9	0.000**
CISS-T	43.9 ± 8.7	42.9 ± 7.1	0.08
CISS-E	46.2 ± 9.1	45.3 ± 7.6	0.07
CISS-A	50.7 ± 9.2	50.5 ± 7.8	0.81

*EAT-26* Eating Attitudes Test-26, *BITE* Bulimic Inventory Test, Edinburgh, *BDI-II* Beck Depression Inventory-II

**p < 0.01

**Table 3:** Female EAT, BITE, BDI, CISS scores at Times 1 and 2

	Time-1	Time-2	*P*-value
	N = 290	N = 290	
EAT-26	3.6 ± 4.9	2.9 ± 4.7	0.051
BITE	5.2 ± 4.2	6.3 ± 5.6	0.000**
BDI-II	5.0 ± 5.7	7.9 ± 7.2	0.000**
CISS-T	43.6 ± 7.2	44.0 ± 6.9	0.89
CISS-E	45.4 ± 8.0	45.5 ± 7.6	0.97
CISS-A	49.6 ± 8.3	48.5 ± 7.8	0.73

*EAT-26* Eating Attitudes Test-26, *BITE* Bulimic Inventory Test, Edinburgh, *BDI-II* Beck Depression Inventory-II

**p < 0.01

Based on these results, the EAT, BITE and BDI scores tend to worsen during college. Therefore, these tools might be useful for identifying at risk students to prevent bulimia and the depressive symptoms.

### Changes in individual BITE scores

**Fig. 1:**
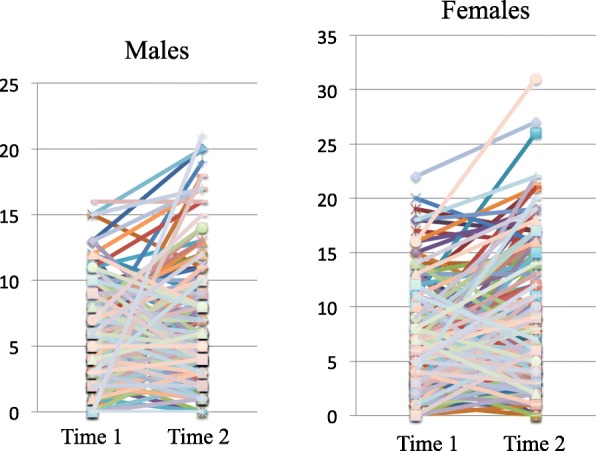
Change in the BITE score of each student. Figure 1 shows the change in the BITE of each student, by sex. Although some students had lower BITE scores, more students had higher BITE score

**Fig. 2:**
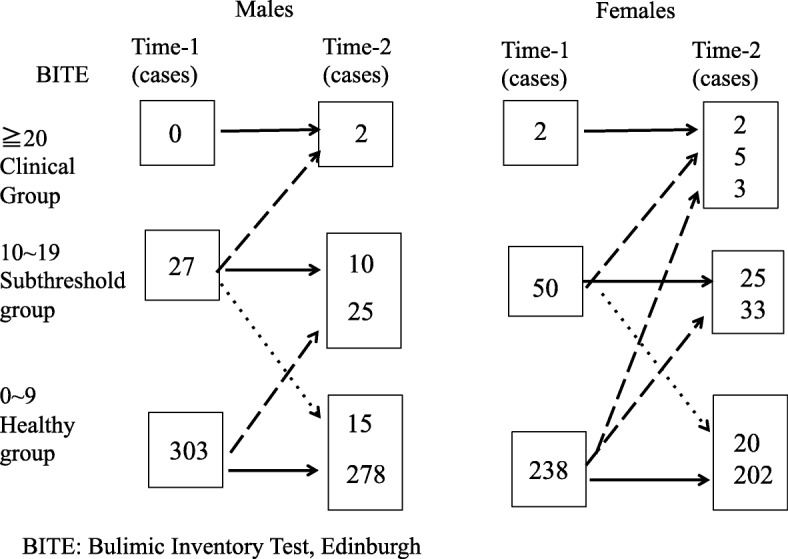
Change in BITE scores. Two males in the subthreshold group shifted to the clinical group, 10 shifted to the subthreshold group, 15 shifted to the healthy group, and 25 in the healthy group shifted to the subthreshold group. Two females in the clinical group remained in this group, 5 in the subthreshold group shifted to the clinical group, 3 in the healthy group shifted to the clinical group, and 33 shifted to the subthreshold group

The changes in the individual BITE scores of the male and female students are shown in Fig. 1. Changes in the clinical, subthreshold, and healthy groups are shown in Fig. 2. More students shifted from the healthy group to the subthreshold group or the clinical group, as evidenced by the increase in the BITE score, than students who showed a reduction in the BITE score. Students in the clinical group remained in the clinical group, with no decrease in the BITE score at Time-2. Approximately half of the males in the subthreshold group showed a reduction in their BITE scores to the levels of the healthy group, whereas 1/8 of females in the subthreshold group shifted to the clinical group, 5/8 remained in the subthreshold group, and 1/4 shifted to the healthy group. In the subthreshold group, fewer individuals showed an improvement in eating behaviors, particularly females; therefore, early intervention for both the subthreshold and clinical groups appears to be necessary.

### Comparison of the BITE i006Dproved (IG), worsened (WG), and unchanged (UG) group BDI-II scores at Time-1

We defined students who changed from the subthreshold group to the healthy group as IG. Students who changed from the healthy group to the subthreshold group, from the subthreshold group to the clinical group, and those remaining in clinical group were designated WG. In addition, we defined students who remained in the healthy group as UG. The BDI-II scores of both males and females in the WG group at Time-1 were significantly higher than the scores of students in the IG and UG groups (Table 4). The higher the level of depression, the more likely that the BITE score worsened. For the males, there was no significant difference between the scores of IG and those of UG. For the females, the scores of IG were significantly higher than those of UG. There was no significant difference between the CISS scores.

**Table 4:** Comparison of BDI-II of the BITE improved, worsened, and unchanged groups

	Improved group	Worsened group	Unchanged group
Male	4.2 ± 3.4	4.7 ± 4.0*	4.3 ± 3.8
	(*N* = 15)	(*N* = 27)	(*N* = 278)
Female	5.3 ± 5.1*	7.6 ± 6.9**	4.8 ± 4.2
	(*N* = 20)	(*N* = 43)	(*N* = 202)

For males, the BDI of the worsened group was significantly higher than those of the improved and unchanged groups

For females, the BDI of the worsened group was significantly higher than those of the improved and unchanged groups, and the BDI of the improved group was significantly higher than that of the unchanged group

*BITE* Bulimic Inventory Test, Edinburgh, *BDI-II* Beck Depression Inventory-II

*p < .05

**p < 0.01

### Comparison of BDI-II and CISS scores at Time-1 between the subthreshold group and the healthy group

Table 5 shows a comparison of the BDI-II and CISS scores at Time-1 between the subthreshold group and the healthy group. Significantly higher BDI-II scores were recorded for males and females in the subthreshold group at Time-1 than for the healthy group. No significant difference in the CISS scores was observed between males in the subthreshold group and the healthy group. However, females in the subthreshold group recorded significantly higher CISS-T and CISS-E scores at Time-1 than females in the healthy group. Thus, the students in the subthreshold group may have tried to objectively cope with stress but were unsuccessful and ultimately used emotional coping strategies.

**Table 5:** Comparison of the BDI-II and CISS of the subthreshold and healthy groups by BITE at Time-1

		Subthreshold group	Healthy group	*P*-value
Male	BDI-II	9.4 ± 7.0	4.0 ± 4.8	0.005**
	CISS-T	43.1 ± 6.4	41.8 ± 7.2	0.454
	CISS-E	46.0 ± 7.2	44.2 ± 7.6	0.995
	CISS-A	50.0 ± 5.6	48.7 ± 8.0	0.087
Female	BDI-II	10.0 ± 9.4	4.1 ± 4.2	0.000**
	CISS-T	46.5 ± 8.4	43.6 ± 6.2	0.046*
	CISS-E	47.5 ± 9.1	45.2 ± 7.2	0.039*
	CISS-A	49.6 ± 7.6	51.2 ± 8.9	0.146

*BITE* Bulimic Inventory Test, Edinburgh, *BDI-II* Beck Depression Inventory-II, *CISS-T* Coping Inventory for Stressful Situations, Task-oriented coping, *CISS-E* CISS Emotion-oriented coping, *CISS-A* CISS Avoidance-oriented coping

*p < 0.05

**p < 0.01

## Discussion

In the present study, we investigated individual changes in students’ scores from Time-1 to Time-2, as well as overall changes in eating attitudes. This strategy is different from previous studies. A greater number of individuals displayed an increased risk of developing bulimia than the number of individuals displaying a decreased risk. In addition, the risk of bulimia did not improve in 3/4 of the individuals, particularly the females in the subthreshold group. This result suggests that preventive intervention is necessary for the subthreshold group. A 5-year longitudinal population-based study reported that one-third of adolescents were asymptomatic at baseline and that more than half of the individuals who were symptomatic at baseline showed symptoms 5 years later [29]. Similar to previous reports, the present study also revealed that problematic eating behaviors persisted once they appeared, and thus early intervention is needed.

The BITE scores increased for both males and females from Time-1 to Time-2, suggesting that bulimic symptoms might worsen during college years. In addition, the EAT-26 score was significantly decreased for males and tended to decrease for females; therefore, in some cases, bulimic symptoms may have developed from anorexic symptoms. Thus, early detection of an increase in the bulimic symptoms of college students is important.

Another 8-year prospective community study of young women found that the onset of BN was 16–20 years old [9]. Based on these survey results, early screening and intervention may be appropriate for college students. According to several studies of college students, the prevalence of eating disorders is high, ranging from 11 to 17% in female students and 4% in male students. A study of 12 colleges in the United States [30] reported that binge eating was observed in 49% of females and 30% of males. In Japan, many college students are estimated to present bulimic symptoms. The increase in the already high eating disorder rate on college campuses combined with academic and social stress, transitional and environmental factors, and a lack of campus resources creates a combination of factors that may result in crisis [31]. In addition, the university is the last educational institution attended before college students enter society; therefore, it is reasonable to screen college students to enable clinicians to provide early intervention for high-risk students.

In terms of an effective early intervention, the developmental pathways of eating disorders should be explored [31]. In the present study, we focused on the association with depressive symptoms. We compared a group showing improved BITE scores from Time-1 to Time-2 with a group whose scores worsened to examine the relation between changes in BITE scores and depressive symptoms. Bulimic symptoms and depressive symptoms worsened in both males and females. The causal risk factors for eating disorder symptoms are important [32–35]. As shown in the study by Pearson et al. [36], adolescents with depressive symptoms that were one standard deviation higher than their peers had a 53% higher risk of transitioning from the asymptomatic group to the disordered group. Depressive symptoms are a risk factor for eating disorders and are specifically associated with worsening bulimic symptoms. The lifetime prevalence of suicidal ideation among patients with BN has been reported to be 15–23% [37] and 26–38% [38]. According to Corcos, patients with BN who had a history of suicide attempts exhibited a higher prevalence of depressive disorders than patients with BN without a suicide attempt history [39]. In the study by Preti, individuals with BN were seven times more likely to die by suicide than females aged 15–34 in the general population [40]. In the early intervention for BN, interventions for both eating behaviors and depressive symptoms may be the key to preventing severe eating disorders and suicide.

In addition, we considered that a focus on the subthreshold group would be important for early intervention; therefore, we compared the subthreshold group with the healthy group. BDI-II scores were significantly higher for both males and females in the subthreshold group than in the healthy group. We cannot conclude that intervention for depressive symptoms would be effective at preventing BN based only on this result, but interventions targeting both eating behaviors and depressive symptoms may be more effective.

Furthermore, the tendency for female students in the subthreshold group to emotionally respond to stress was related to disordered eating. Therefore, stress coping interventions may be important for the prevention of disordered eating. There have been reports on eating disorders and stress coping [41–45]. Some reports have examined the relation between eating disorders and avoidance coping behaviors [42], particularly AN [43]. On the other hand, patients with BN display a significantly increased use of emotional coping behaviors [34, 45]. We speculate that AN may be associated with avoidance coping behaviors and BN with emotional coping behaviors. In terms of early intervention, cognitive behavioral approaches designed to improve coping skills and resolve problems objectively during stressful situations are thought to be effective.

Previous studies have reported that sexual minorities [46] and athletes [47, 48] are at a high risk of developing BN. In the present study, we did not investigate sexual minorities. In terms of athletes, we did not observe a difference in the students’ departments (major). In addition, the student’s living environment (living with family or living alone) can also be related to BN, but we did not consider this factor in the present study.

### Limitations

A limitation of this study is that it only considered depressive symptoms and stress coping behaviors as risk factors. Self-esteem, personality tendencies, and other mental symptoms should also be considered to identify more risk factors. Furthermore, the number of subjects decreased by 50% from Time-1 to Time-2, because the number of students who underwent a medical examination in the 4th year (Time-2) decreased. The reason for this decrease is because medical examinations are not compulsory, and thus few students underwent a medical examination unless it was required when they were applying for jobs. Although not shown in the results, the BITE scores of students who did not receive a medical examination at Time-2 did not differ from the participants. However, some bias may have occurred. In addition, the assessment was a self-rated questionnaire. In the future, more accurate information and a diagnosis must be obtained through a direct interview.

This study was conducted at a single campus, thereby limiting the generalizability of the findings. More survey results should be obtained in the future in collaboration with other colleges in Japan.

### Future work

In the future, we would like to increase the number of subjects and identify additional candidate risk factors. By examining subjects for a longer follow-up period, we will be better able to identify risk factors and develop intervention methods.

## Conclusions

Depressive symptoms are a risk factor for eating disorders and are specifically associated with worsening bulimic symptoms. Interventions for depressive symptoms and stress coping may be key factors in early intervention for bulimia. Furthermore, the results suggest that early intervention may be necessary for both the subthreshold and clinical groups.

## Abbreviations

AN: Anorexia nervosa
BDI-II: Beck depression inventory-II
BITE: Bulimic inventory test, Edinburgh
BN: Bulimia nervosa
CISS: Coping inventory for stressful situations
EAT-26: Eating attitudes test-26

## References

[1] Jacobi C, Paul T, de Zwann M, Nutzinger DO, Dahme B (2004). Specificity of self-concept disturbances in eating disorders. Int J Eat Disord..

[2] Crow SJ, Peterson CB, Maj M, Halmi KA, Lopez-Ibor JJ, Sartrius N (2003). The economic and social burden of eating disorders. In WPA series evidence and experience in psychiatry. Eating Disorders.

[3] Crow SJ, Agra WS, Halmi K, Mitchell JE, Kraemer HC (2012). Full syndromal versus subthreshold anorexia nervosa, bulimia nervosa, and binge eating disorder: a multicenter study. Int J Eat Disord..

[4] Crow SJ, Peterson CB, Swanson SA, Raymond NC, Specker S, Eckert ED, Mitchell JE (2009). Increased mortality in bulimia nervosa and other eating disorders. The Am J Psychiaty.

[5] Pisetsky EM, Peterson CB, Mitchell JE, Wonderlich SA, Crosby RD, LeGrange D, Hill L, Powers P, Crow SJ. A comparison the frequency of familial suicide attempts across eating disorder diagnosis. Int J Eat Disord. 2017;15 10.1002/eat.22694. 10.1002/eat.22694 PMC5459656 28199032

[6] Pisetsky EM, Haynos AF, Lavender JM, Crow SJ, Peterson CB. Associations between emotion regulation difficulties, eating disorder symptoms, non-suicidal self-injury, and suicide attempts in a heterogeneous eating disorder sample. Compr Psychiatry 2017;73:143–150 doi:10.1016/j.coppsych.2016.11.012. 10.1016/j.comppsych.2016.11.012 PMC5263187 27978502

[7] Striegel Weissman R, Rosselli F (2017). Reducing the burden of suffering from eating disorders: unmet treatment needs, cost of illness, and the quest for cost-effectiveness. Behav Res Ther.

[8] Anastasiadou D, Parks M, Brugnera A, Sepulveda AR, Graell M (2016). Psychiatric comorbidity and maternal distress among adolescent eating disorder patients: a comparison with substance use disorder patients. Eat Behav.

[9] Stice E, Marti CN, Rohde P (2013). Prevalence, incidence, impairment, and course of the proposed DSM-5 eating disorder diagnosis in 8-year prospective community study of young women. J Abnorm Psychol.

[10] Nagl M, Jacobi C, Paul M, Beesdo-Baum K, Höfler M, Lieb R, Wittchen H (2016). Prevalence, incidence, and natural course of anorexia and bulimia nervosa among adolescents and young adults. Eur Child Acolesc Psychiatry.

[11] Takagaki K, Okamoto Y, Jinnin R, Mori A, Nishiyama Y, Yamamura T, Yokoyama S, Shiota S, Okamoto Y, Miyake Y, Ogata A, Kunisato Y, Shimoda H, Kawakami N, Furukawa TA, Yamawaki S (2016). Behavioral activation for late adolescents with subthreshold depression: a randomized control trial. Eur Child Adolesc Psychiatry.

[12] Hashimoto N, Suzuki Y, Kato TA, Fujisawa D, Sato R, Aoyama-Uehara K, Fukasawa M, Asakura S, Kusumi I, Otsuka K (2016). Effectiveness of suicide prevention gatekeeper-training for university staff in Japan. Psychiaty Clin Neurosci.

[13] Uchida C, Uchida M (2017). Characteristics and risk factors for suicide and deaths among college students: a 23-year serial prevalence study of data from 8.2 million Japanese college students. J Clin Psychiatry.

[14] Mills JS, Polivy J, McFarlane TL, Crosby RD (2012). The natural course of eating pathology in female university students. Eat Behav.

[15] Gropper SS, Arsiwalla DD, Lord DC, Huggins KW, Simmons KP, Ulrich PV (2014). Associations among eating regulation and body mass index, weight, and body fat in college students: the moderating role of gender. Eat Behav.

[16] Prouty AM, Protinsky HO, Canady D (2002). College women: eating behaviors and help-seeking preferences. Adolescence.

[17] Eisenberg D, Nicklett EJ, Roeder K, Kirz NE (2011). Eating disorder symptoms among college students: prevalence, persistence, correlates, and treatment-seeking. J Am Coll Heal.

[18] Taylor JV, Gibson DM (2016). Crisis on campus: eating disorder intervention from a developmental-ecological perspective. J Am Coll Heal.

[19] Lipson SK, Sonneville KR (2017). Eating disorder symptoms among undergraduate and graduate students at 12 U.S. colleges and universities. Eat Behav.

[20] Stice E, Marti CN, Shaw H, Jaconis M (2009). An 8-year longitudinal study of the natural history of threshold, subthreshold, and partial eating disorders from a community sample of adolescents. J Abnorm Psychol.

[21] Keski-Rahkonen A, Mustelin L (2016). Epidemiology of eating disorders in Europe: prevalence, incidence, comorbidity, course, consequences, and risk factors. Curr Opin Psychiatry.

[22] Brand-Gothelf A, Leor S, Apter A, Fennig S (2014). The impact of comorbid depressive and anxiety disorders on severity of anorexia nervosa in adolescent girls. J Nerv Ment Dis.

[23] Okamoto Y, Nakatsu H, Kawamura T (2000). Mood states and stress coping of patients with eating disorders in relation to short-term intervention. Japanese journal of. Psychosom Med.

[24] Okamoto Y, Miyake Y, Nagasawa I, Shishida K (2017). A 10-year follo-up study of completers versus dropouts following treatment with an integrated cognitive-behavioral group therapy for eating disorders. J Eat Disord.

[25] Garner DM, Olmsted MP, Bohr Y, Garfinkel PE (1982). The eating attitudes test: psychometric features and clinical correlates. Psychol Med.

[26] Henderson M, Freeman CP (1987). A self-rating scale for bulimia. The ‘BITE’. Br J Psychiatry.

[27] Beck AT, Ward CH, Mendelson M, Mock J, Erbaugh J (1961). An inventory for measuring depression. Arch Gen Psychiatry.

[28] Endler NS, Parker JDA (1990). Coping inventory for stressful situations (CISS): manual.

[29] Ackard DM, Fulkerson JA, Neumark-Sztainer D (2011). Stability of eating disorder diagnostic classifications in adolescents: five-year longitudinal findings from a population-based study. Eat Disord.

[30] Lipson SK, Sonneville KR (2017). Eating disorder symptoms among undergraduate students at 12 U.S. colleges and universities. Eat Behav.

[31] Le Grange D, O’Connor M, Hughes EK, Macdonald J, Little K, Olsson CA (2014). Developmental antecedents of abnormal eating attitudes and behaviors in adolescence. Int J Eat Disord..

[32] Stice E, Hayward C, Cameron R, Killen JD, Taylor CB (2000). Body image and eating disturbances predict onset of depression in female adolescents: a longitudinal study. J Abnorm Psychol.

[33] Field AE, Austin SB, Taylor CB, Malspeis S, Rosner B, Rockett HR, Gillman MW, Colditz GA (2003). Relation between dieting and weight change among preadolescents and adolescents. Pediatrics and Neonatology.

[34] Chamay-weber C, Narring F, Michaud P (2005). Partial eating disorders among adolescents: a review. J Adolesc Health.

[35] Combs JL, Pearson CM, Zapolski TCB, Smith GT (2012). Preadolescent disordered eating predicts subsequent eating dysfunction. J Pediat Psychol.

[36] Pearson CM, Miller J, Ackard DM, Loth KA, Wall MM, Haynos AF, Neumark-Sztainer D. Stability and change in patterns of eating disorder symptoms from adolescence to young adulthood. Intl J Eat Disord. 2017; 10.1002/eat.22692. 10.1002/eat.22692 PMC5505795 28199037

[37] Favao A, Santonastaso P (1997). Suicidality in eating disorders: clinical and psychological correlates. Acta Psychiatr Scand.

[38] Milos G, Spindler A, Hepp U, Schnyder U (2004). Suicide attempts and suicidal ideation: link with psychiatric comorbidity in eating disorder subjects. Gen Hosp Psychiatry.

[39] Corcos M, Taieb O, Benoit-Lamy S, Paterniti S, Jeammet P, Flament MF (2002). Suicide attempts in women with bulimia nervosa: frequency and characteristics. Acta Psycchiatr. Scandinavica.

[40] Preti A, Rocchi MBL, Sisti D, Cambini MV, Miotto P (2011). A comprehensive meta-analysis of the risk of suicide in eating disorders. Acta Psychiatr Scand.

[41] Costarelli V, Patsai A (2012). Academic examination stress increase disordered eating symptomatology in female university students. Eat Weight Disord.

[42] Turner H, Bryant-Waugh R, Peveler R (2009). An approach to sub-grouping the eating disorder population: adding attachment and coping style. Eur Eat Disord Rev.

[43] Macneil L, Esposito-Smythers C, Mehlenbeck R, Weismoore J (2012). The effects of avoidance coping and coping self-efficacy on eating disorder attitudes and behaviors: a stress-diathesis model. Eat Behav.

[44] Nagata T, Matsuyama M, Kiriike N, Iketani T, Oshima J (2000). Stress coping strategy in Japanese patients with eating disorders: relationship with bulimic and impulsive behaviors. J Nerv Ment Dis.

[45] Clyne C, Latner JD, Gleaves DH, Blampied NM (2010). Treatment of emotional dysregulation in full syndrome and subthreshold binge eating disorder. Eat Disord.

[46] Mathews-Edward MR, Zullig KJ, Ward RM (2014). Sexual orientation and disordered eating behaviors among self-identified male and female college students. Eat Behav.

[47] DiPasquale LD, Petrie TA (2013). Prevalence of disordered eating: a comparison of male and female collegiate athletes and nonathletes. J Clin Sport Psychol.

[48] Wollenberg G, Shriver LH, Gates GE (2015). Comparison of disordered eating symptoms and emotion regulation difficulties between female college athletes and non-athletes. Eat Behav.

